# Supplementary material for: The integration of communicable and non-communicable disease (CD-NCD) health services in Africa: A scoping review
Source: PLOS Glob Public Health. 2026 Mar 26;6(3):e0006087. doi: 10.1371/journal.pgph.0006087 (PMC13020777; doi:10.1371/journal.pgph.0006087)
Supplement: S1 Appendix – — (DOCX) [file pgph.0006087.s001.docx]

**S1 Appendix:**

| **PubMed Search String** |
| --- |
| ((("HIV"[tiab] OR "HIV/AIDS"[tiab] OR "Tuberculosis"[tiab] OR "TB"[tiab] OR "Malaria"[tiab] OR "HPV"[tiab] OR "chlamydia"[tiab] OR "syphilis"[tiab] OR "herpes"[tiab] OR "gonorrhea"[tiab] OR "Hepatitis B"[tiab] OR "Hepatitis C"[tiab] OR "Schistosomiasis"[tiab] OR "Cholera"[tiab]) AND ("Cancer"[tiab] OR "Diabetes"[tiab] OR "Heart disease"[tiab] OR "Hypertension"[tiab] OR "high cholesterol"[tiab] OR "Cardiovascular disease"[tiab] OR "hypercholesterolemia"[tiab] OR "Chronic obstructive pulmonary disease"[tiab] OR "Asthma"[tiab] OR "Chronic kidney disease"[tiab] OR "Liver disease"[tiab] OR "Stroke"[tiab] OR "Mental health disorders"[tiab] OR "Depression"[tiab] OR "Alzheimer's disease"[tiab])) AND ("Systems Integration"[tiab] OR "Integrated care"[tiab] OR "Coordinated care"[tiab] OR "Collaborative care"[tiab] OR "Chronic care model*"[tiab] OR "integrated delivery system"[tiab] OR "disease management"[tiab] OR "case management"[tiab] OR "patient care management"[tiab] OR "patient-centered care"[tiab] OR "managed care"[tiab] OR "comprehensive care"[tiab] OR "seamless care"[tiab] OR "continuity of care"[tiab] OR "patient care planning"[tiab] OR "patient care team"[tiab] OR "medical home"[tiab])) AND ("Africa"[tiab] OR "sub-Saharan Africa"[tiab] OR "Algeria"[tiab] OR "Angola"[tiab] OR "Benin"[tiab] OR "Botswana"[tiab] OR "Burkina Faso"[tiab] OR "Burundi"[tiab] OR "Cape Verde"[tiab] OR "Cameroon"[tiab] OR "Central African Republic"[tiab] OR "Chad"[tiab] OR "Comoros"[tiab] OR "Democratic Republic of the Congo"[tiab] OR "Republic of the Congo"[tiab] OR "Djibouti"[tiab] OR "Egypt"[tiab] OR "Equatorial Guinea"[tiab] OR "Eritrea"[tiab] OR "Eswatini"[tiab] OR "Ethiopia"[tiab] OR "Gabon"[tiab] OR "Gambia"[tiab] OR "Ghana"[tiab] OR "Guinea"[tiab] OR "Guinea-Bissau"[tiab] OR "Ivory Coast"[tiab] OR "Kenya"[tiab] OR "Lesotho"[tiab] OR "Liberia"[tiab] OR "Libya"[tiab] OR "Madagascar"[tiab] OR "Malawi"[tiab] OR "Mali"[tiab] OR "Mauritania"[tiab] OR "Mauritius"[tiab] OR "Morocco"[tiab] OR "Mozambique"[tiab] OR "Namibia"[tiab] OR "Niger"[tiab] OR "Nigeria"[tiab] OR "Rwanda"[tiab] OR "Sao Tome and Principe"[tiab] OR "Senegal"[tiab] OR "Seychelles"[tiab] OR "Sierra Leone"[tiab] OR "Somalia"[tiab] OR "South Africa"[tiab] OR "South Sudan"[tiab] OR "Sudan"[tiab] OR "Tanzania"[tiab] OR "Togo"[tiab] OR "Tunisia"[tiab] OR "Uganda"[tiab] OR "Zambia"[tiab] OR "Zimbabwe"[tiab]) |
