# Supplementary material for: The integration of communicable and non-communicable disease (CD-NCD) health services in Africa: A scoping review
Source: PLOS Glob Public Health. 2026 Mar 26;6(3):e0006087. doi: 10.1371/journal.pgph.0006087 (PMC13020777; doi:10.1371/journal.pgph.0006087)
Supplement: S2 Appendix – — (DOCX) [file pgph.0006087.s002.docx]

**S2 Appendix:**

Chat showing use of large language model for text categorization and frequency counting: <https://chatgpt.com/share/6863fc00-9260-800f-9343-f089220836c1>
