# Supplementary material for: The integration of communicable and non-communicable disease (CD-NCD) health services in Africa: A scoping review
Source: PLOS Glob Public Health. 2026 Mar 26;6(3):e0006087. doi: 10.1371/journal.pgph.0006087 (PMC13020777; doi:10.1371/journal.pgph.0006087)
Supplement: Attachment — Submitted filename: Response to reviewers-Jan12.docx [file pgph.0006087.s005.docx]

January 12, 2025

Dr. Jen Edwards

Staff Editor

PLOS Global Public Health

Dear Dr. Jen Edwards,

Thank you for giving us the opportunity to submit a revised draft of our manuscript titled **The integration of communicable and non-communicable disease (CD-NCD) health services in Africa: A Scoping Review** to *PLOS Global Public Health.* We appreciate the time and effort that you and the reviewer have dedicated to providing valuable feedback on our manuscript.

Attached to this letter you will find point-by-point responses to comments from the editorial staff and the reviewer. The suggested changes have led to an improved manuscript and pushed this work to be both more precise and relevant to interested readers. We are grateful for the reviewer’s feedback. The main changes were:

- We have defined the scope of the paper to be more specifically about the combined provision of communicable and non-communicable disease health services at a single point of delivery, which we contrast with single-disease programs.
- We have included additional discussion on different conceptualizations of *integration* and the need for more non-disease-centric but patient-centered approaches, as elucidated by the studies found in our review of the literature.

The point-by-point responses provide more details about these changes. We look forward to discussing this research with the editorial team. Thank you very much for your consideration of our revised manuscript and response to the reviewer in this resubmission.

Best regards,

Takhona Hlatshwako, MSc

PhD Student, Health Policy and Management

University of North Carolina at Chapel Hill

**Comments from Reviewer 1**

1. **Comment:** The first critique is that I think the paper falls short by not offering a more critical assessment of the construct of "integration" and how it is used in the studies cited in this review.

There are at least two different ways one could conceptualize integration. One is to look holistically at different health system functions and the pros and cons of using vertical/targeted approaches to deliver certain services. A couple of articles by Atun, Adeyi, and colleagues from 2010 in Health Policy and Planning summarize this way of thinking. The other is to focus on "adding on" new services to a set of existing services, often for a given (priority) patient population. Much of the literature around HIV has gone this direction - for example, see a July 2018 supplement in AIDS. This scoping review aligns implicitly with the latter, and as expected, the literature uncovered in this scoping review almost exclusively focuses on adding services for PLHIV with non-HIV health needs.

While there is nothing wrong with improving primary care for PLHIV, this conceptualization of integration falls massively short as a way of guiding health system development.

**Response:** Thank you for bringing forth this important point. We have revised the manuscript by adding a clearer definition of “an integrated care model” which we define as “as the combined provision of health services for both communicable and non-communicable diseases at a single point of delivery” (this definition aligns with the reviewer’s second suggested definition). Further, we include a counterfactual to further clarify the construct on integration: “We conceptualized the alternative as a narrow-focused program providing services for a single disease, such as an HIV clinic” (Page 6, line 121). While we agree that this definition does not encapsulate changes to an entire health system, the goal of health system development is beyond the scope of our paper. Primarily, we sought to offer a descriptive account of the tangible ways health services have been combined for more innovative delivery across the continent, which as we note in our introduction, several nations have developed action plans with the intent to do so. Nonetheless, we have included further commentary on the need for more nuanced views of integration in the “implications” section of the paper, particularly pointing out that “There is a need for a more robust and nuanced view of *integration* to guide broader health system innovation and move us towards more holistic, patient-centered care at all levels. Particularly, researchers, policymakers and practitioners ought to consider integrated models that move beyond simply adding more health services but rather transform healthcare delivery to better align with community needs.” (Page 20, line 604)

**We have also referenced the suggested paper by Atun and colleagues to demonstrate how our assessment of integration fits into the wider discourse on this topic:**

“Moreover, several scholars have emphasized the need for a more nuanced conceptualization of “integration,” particularly noting that the binary contrast of horizontal and vertical programs is a limited view. For instance, a conceptual framework for integration by Atun and colleagues proposes several facets that can be used to deconstruct integration according to functions served in the health system. Under this framework, the interventions described by our review fall within the ‘health service delivery’ function of the health system, specifically at the initial stage of combining services that are perceived as more efficiently addressed together, such as cardiometabolic issues (diabetes and hypertension, for instance). However, there are several steps beyond combination of services that are necessary for true whole health system transformation i.e., adaptation, evaluation, scale-up, etc. Notably, most of the studies noted in our review were most often small-scale interventions, some experimental, and limited to one or two health facilities at most, demonstrating that there is still a long road ahead to achieve health system integration and transformation.” (Page 18, line 520)

2. **Comment:** PLHIV are indeed a priority population group, but they only represent 2.5% of the population, and they have been fortunate to have a lot of resources put into developing their own healthcare system that largely operates in parallel to the general healthcare system. What about the other 97.5% of the population?

**Response:** We agree that there is a larger portion of the population that is often left out in integration discourse. As such, we specifically did not restrict our population of interest to PLHIV in our search for this very reason, and we searched for a broad range of communicable and non-communicable diseases. For instance, we found an integrated model for Diabetes and Tuberculosis (Table 1). However, as HIV is still a major challenge in many African countries –indeed in [some countries](https://data.worldbank.org/indicator/SH.DYN.AIDS.ZS?locations=SZ-LS-ZA-BW) the population living with HIV is 15-20%-- most of the literature we found reported on integrated models including HIV. Nonetheless, we have emphasized the need for broader types of care delivery models in our discussion section (Page 17-18, line 500).

3. **Comment:** What is needed, I think, is an overhaul of pre-service training of PHC nurses so that they have the skills needed to do basic chronic disease screening and treatment. And over the long term, national governments need to invest in the family medicine PHC model, where one primary care provider (doctor or advanced-practice provider) is able to do first-line evaluation and management of pretty much any health concern a patient comes in with…

**Response:** We agree that primary care providers are crucial for integrated care models. We have revised our discussion section to further include these suggestions for policymakers and health system decision-makers to consider, along with a reference (page 19, line 564).

4. **Comment:** Put another way, we're having this conversation about "integration" in African countries because we're still dealing with the legacy of colonialism and neo-colonial ideas about how a health system should be designed (and which patients should receive priority for services). I think we need to be pushing researchers to think about integration in a much broader way than simply teaching ART nurses to screen for hypertension.

I don't expect the authors to solve this problem, or to change the substance of their review. But I think we need to be slowly shifting the discourse around integration to focus on patient-centered care for everyone, rather than making tweaks to donor initiatives….

**Response.** Agreed. We have revised our discussion to include this important point as referenced earlier (Page 20, line 605). Particularly, we have included a commentary on “authentic allyship,” stressing the need for an evolved approach to funding research and programs towards health system transformation: “Funders of research and programs play a key role in this endeavor through working to reduce power asymmetries and pursuing what others have called *authentic allyship.* Indeed, to enhance health system transformation in nations across the continent, true partnerships focused on demonstrated need, rigorous science, and community-led wisdom will be critical.” (Page 20, line 608)

5. **Comment:** For this scoping review, I would hope for a few additional sentences here and there in the results and discussion sections that underscore these following points (assuming the authors agree):

(1) there are different ways of thinking about integration (see my first point above);

(2) the "model types" that the authors have uncovered (Table 1) are mostly focused on adding services one- or two-at-a-time into HIV care, and these still need to be integrated amongst themselves (!); and

(3) this heavy focus on incremental improvements in care for PLHIV has led to major blind spots in the literature about how to develop PHC systems that provide essential services for the entire population.

**Response:** Agreed. Thank you for these suggestions. We have included these points:

1) Integrated care models vary widely, and *integration* may be conceptualized in a variety of ways, such as an innovation to health service delivery, organization, financing, and other health system functions. In particular, integrated care models for health service delivery — the focus of this review— are often characterized by several key features, including coordination and collaboration among healthcare providers, communication across different healthcare levels, and a patient-centered focus to ensure overall well-being. (Page 5, line 83)

While there are numerous conceptualizations of integrated care, we focused on models that combined or consolidated health services for both communicable and non-communicable diseases (Page 16, line 441)

2) Particularly, researchers, policymakers and practitioners ought to consider integrated models that move beyond simply adding more health services but rather transform healthcare delivery to better align with community needs. (Page 20, line 606)
3) Another study of TB-diabetic patients in Tanzania reported on the urgent need for integrated care models for patients suffering from TB and diabetes comorbidity, which is evidence of the need to expand on current HIV-based models to include other health concerns in the population. (Page 18, line 510)

Additionally, although we have focused on a specific definition of integration of health services, there is minimal research on this topic –despite demonstrated interest by governments, as cited in our paper - that offers guidance on how to do this kind of integration. Moreover, we believe our review also reveals what has not been studied, and the need for more research in this area.

6. **Comment:** The second critique is that I'm not sure if a disease or risk factor constitutes a "model" per se (as per Table 1). I think models of care are better defined around human resources (eg, competencies/scope of practice) and the geospatial arrangement of different components of care (eg, dispensaries vs. health centers vs. hospitals).

**Response:** Thank you for this important point. As mentioned earlier, we sought to provide a descriptive account of the ways integrated care has been deployed on the continent. Towards this end, we believe presenting the disease areas of focus for each integrated model to be the most easily understood and accessible way of presenting our findings to a broad range of readers and stakeholders. Additionally, this is the way most of the studies in our review presented their interventions, as “integrated models.” Moreover, our knowledge of the full scope of human resources and geospatial arrangements of each model is limited only to what is recorded in each study, and the level of depth across the studies (as captured by our quality assessment) varies. Therefore, we believe such a categorization would be incomplete. Nonetheless, the discernible characteristics of each integrated care model are described in Table 2 to provide further details of the features of the different models we found, which we hope is sufficient.

7. **Comment:** There is no explicit mention of a grey literature search. I could imagine some reports on NGO websites or other parts of the internet on real-world care delivery that were never written up as peer-reviewed papers.

**Response:** Agreed. Grey literature was searched to equip the authors with relevant background information on integrated care models and their structure. However, for this review, we decided to search and include only peer-reviewed literature to ensure a robust report of findings.

8. **Comment:** The English language restriction should be defended more than it is currently. What proportion of the literature is potentially being missed by not considering studies (including, importantly, in the grey literature) from francophone, arabophone, and lusophone countries?

**Response:** Thank you for pointing this out. We have clarified in our paper that enforcing the language restriction did not exclude any papers in our search (Page 6, line 142). We have also added this statement to our limitations section: “We limited our review to peer-reviewed papers written in the English language and used mostly English-based databases. Although our language restriction to English did not exclude any peer-reviewed papers in the databases we searched, we did not search any literature in other languages. Future reviews may benefit from searching non-English literature, as well as grey literature.” (page 20, line 588)

9. **Comment:** The paper mentions "African region" but does not define the countries referred to. Do they mean sub-Saharan Africa, WHO-AFRO, or something else? The appendix search strategy implies AFRO, but it would be good to say this in the main text.

**Response:** Thank you for pointing this out. We have clarified that we mean the whole African region (North and Sub-Saharan Africa) (Page 6, line 134). The list of countries is in the appendix.

10. **Comment:** Finally, I note that the barriers (and to a lesser extent, enablers) listed in these studies (Table 3) are pretty generic and could describe health system gaps in general, not just CD-NCD care. There's been a gazillion studies on barriers for various diseases, with a lot of overlap. A brief nod to this literature, at least as relates to chronic disease, would be useful.

**Response:** Thank you for pointing this out. We have further described the barriers and facilitators for CD-NCD care. We have also included further references in the discussion section to reflect broader literature on this topic (line 488).
