# Supplementary material for: The integration of communicable and non-communicable disease (CD-NCD) health services in Africa: A scoping review
Source: PLOS Glob Public Health. 2026 Mar 26;6(3):e0006087. doi: 10.1371/journal.pgph.0006087 (PMC13020777; doi:10.1371/journal.pgph.0006087)
Supplement: Attachment — Submitted filename: Response to reviewers-Feb4.docx [file pgph.0006087.s006.docx]

February 4, 2026

Dr. Andrew Kazibwe

Academic Editor

PLOS Global Public Health

Dear Dr. Andrew Kazibwe,

Thank you for giving us the opportunity to submit a revised draft of our manuscript titled **The integration of communicable and non-communicable disease (CD-NCD) health services in Africa: A Scoping Review** to *PLOS Global Public Health.* We appreciate the time and effort that you and the reviewer have dedicated to providing valuable feedback on our manuscript.

Attached to this letter you will find point-by-point responses to comments from the editorial staff and the reviewer. The suggested changes have led to an improved manuscript and pushed this work to be both more precise and relevant to interested readers. We are grateful for the reviewer’s feedback. The main changes were:

- We have clarified the search methodology on how we sought for interventions combining communicable and non-communicable disease health services.
- We completed all necessary revisions to the formatting of figures, financial disclosures, funding source information, and our data availability statement.

The point-by-point responses provide more details about these changes. We look forward to discussing this research with the editorial team. Thank you very much for your consideration of our revised manuscript and response to the reviewer in this resubmission.

Best regards,

Takhona Hlatshwako, MSc

PhD Student, Health Policy and Management

University of North Carolina at Chapel Hill

**Comments from Editorial Staff**

1. Please provide a detailed online Financial Disclosure statement. This is published with the article. It must therefore be completed in full sentences and contain the exact wording you wish to be published.

a) State the initials, alongside each funding source, of each author to receive each grant. For example: “This work was supported by the National Institutes of Health (####### to AM; ###### to CJ) and the National Science Foundation (###### to AM).”

b) State what role the funders took in the study. If the funders had no role in your study, please state: “The funders had no role in study design, data collection and analysis, decision to publish, or preparation of the manuscript.”

For more information, please go to our submission guidelines:
<https://journals.plos.org/globalpublichealth/s/submission-guidelines#loc-financial-disclosure-statement>

We now include the requested Financial Disclosure statement, which we styled after other papers recently published in PLOS Global Public Health. Please find the Financial Disclosure in the manuscript text as well as reproduced below:

TH received support from the Royster Society of Fellows. TT received support from P2CHD050924. OA, JC, LJ, SS, AY, and LF received no specific funding for this work. TT received support from 5R01HD101453-05 and P2CHD050924. The funders had no role in study design, data collection and analysis, decision to publish, or preparation of the manuscript.

2. Please ensure that the funders and grant numbers match between the Financial Disclosure field and the Funding Information tab in your submission form. Note that the funders must be provided in the same order in both places as well.

Thank you we have reviewed the Financial Disclosure field and the Funding Information tab in the submission form for alignment. We have confirmed that the listed funders are in the same order in both places.

3. Please update your online Competing Interests statement. If you have no competing interests to declare, please state: “The authors have declared that no competing interests exist.”

Thank you for this reminder. Our Competing Interests statement is now in the submission portal and in the manuscript as follows: “The authors have declared that no competing interests exist.”

4. We note that your Data Availability Statement is currently as follows: "The data was compiled from published studies. The protocol was registered in OSF (https://doi.org/10.17605/OSF.IO/ZMR5K)."

Please confirm at this time whether or not your submission contains all raw data required to replicate the results of your study. Authors must share the “minimal data set” for their submission. PLOS defines the minimal data set to consist of the data required to replicate all study findings reported in the article, as well as related metadata and methods (https://journals.plos.org/globalpublichealth/s/data-availability#loc-minimal-data-set-definition).

Thank you for this reminder. We have confirmed that all necessary components to replicate our analysis can be found at the OSF repository and within the manuscript and supplementary files (Search Strategy).

5. Please include a separate legend or caption for Figure 1 in your manuscript.

Thank you for this reminder. We now include a separate caption for Figure 1 in the manuscript and it reads as follows: “Figure 1: PRISMA flowchart of included studies”.

6. If the reviewer comments include a recommendation to cite specific previously published works, please review and evaluate these publications to determine whether they are relevant and should be cited. There is no requirement to cite these works unless the editor has indicated otherwise.

We appreciate this comment by the editorial team. We reviewed a piece recommended by Reviewer 1 and determined that it was appropriate to include due to the definition of health care service integration provided.

7. Please review your reference list to ensure that it is complete and correct. If you have cited papers that have been retracted, please include the rationale for doing so in the manuscript text, or remove these references and replace them with relevant current references. Any changes to the reference list should be mentioned in the rebuttal letter that accompanies your revised manuscript. If you need to cite a retracted article, indicate the article’s retracted status in the References list and also include a citation and full reference for the retraction notice.

Thank you for this reminder. We have confirmed that the reference list is complete and correct. We did not find any articles that had been retracted when we reviewed on February 3, 2026.

**Comments from Reviewer 1**

1. Comment: All comments have been addressed

**Response**: We thank the reviewer for reading our work and providing positive and constructive feedback, which improved the manuscript. Thank you for your time and expertise to review our work.

**Comments from Reviewer 2**

1. **Comment**: This is a timely and exciting article. Below are a few comments for consideration:

**Response**: We thank the reviewer for reading our work and providing positive and constructive feedback. We agree that this topic is a timely and important one for the field of global public health. We appreciate the feedback to improve this paper. We have made revisions in response to this review.

2. **Comment:** Whereas the authors aimed to study communicable and non-communicable disease integration, their search seems to have yielded results pertinent to HIV care only, in the communicable disease domain. It is therefore prudent that the study title and introduction reflect this bias (which is justified by the significant cumulative investment in HIV care systems). Other communicable diseases where integration has been key include viral hepatitis, tuberculosis, malaria and bacterial sexually transmitted infections among others. The fact that these did not feature in the article negates the broad focus on communicable diseases as portrayed in the title and background sections.

**Response:** Thank you for this thoughtful comment. We agree that integrated service delivery in practice has indeed focused on many communicable diseases, e.g. this website from the WHO describing integrated services for tuberculosis (<https://www.who.int/europe/activities/implementing-effective-tuberculosis-service-delivery-systems>). Our review question, inclusion criteria, and search strategy were pre-registered to examine integration across communicable and non-communicable disease services broadly. The fact that the resulting peer-reviewed evidence base is overwhelmingly concentrated in HIV care – despite other investments, though modest in comparison, for other communicable diseases—is a result of the review rather than a limitation. We believe this is an important finding in and of itself, and we have included commentary on this finding in the discussion section. To draw attention to this point, we now state: “Although our review was explicitly designed to examine integration of communicable and non-communicable disease services, the near-exclusive focus of identified models on HIV care –despite well-documented integration efforts for tuberculosis, malaria, and other infectious diseases– reveals a substantive gap in the empirical, peer-reviewed literature. We believe this to be an important finding of this review.” (Line 512)

As such, we believe maintaining the title as “communicable and non-communicable health services,” remains true to the intent of the review, and the main finding –that most studies continue to focus on HIV—is an important finding to distinguish.

3. **Comment: There is a discrepancy in the numbers of articles retrieved and included in the protocol referenced, and those reported in the review. These ought to be harmonised, and any discrepancies explained.**

**Response:** Thank you for pointing this out. We conducted the first search in February 2025 on PubMed (which is documented in the protocol), and then replicated the same search in June 2025 after the protocol was published but before submission to ensure that we did not miss any important papers that may have been published in the interim and in other key databases. We have now clarified in the manuscript that we conducted two searches and the noted the dates of the searches as such:

“Key search terms for each concept were identified (Appendix A). We used these terms to search PubMed on February 22, 2025 (documented in the protocol), and then replicated the search on PubMed, MEDLINE, Cochrane Library, SCOPUS, and PsycINFO databases on June 6, 2025 (final cut-off date).” (Line 127)

4. **Comment:** What was the basis for labeling the integration models based on disease entities, rather than service locations?

**Response:** Thank you for this question. We labeled the integration models based on how the studies themselves labeled these models. We believed this to be the most appropriate way to display the findings and maintain consistency with previous literature.

5. **Comment:** In the methodology section, the authors should clearly describe what data was extracted from each source.

**Response:** Thank you for this important point. We have clarified this in the manuscript as such:

“Data extraction was conducted by two independent reviewers, who each extracted the following data from each study: author, year of publication, country, study design, disease areas, number of total participants, intervention components, process outcomes, barriers, facilitators, and the use of digital technology (which we operationalized as both an intervention component and facilitator). The reviewers used the codebook described in the review protocol.” (Line 155)

We thank the reviewer for the constructive feedback. It is very appreciated and we believe that the feedback has improved the paper.
