# Supplementary material for: The integration of communicable and non-communicable disease (CD-NCD) health services in Africa: A scoping review
Source: PLOS Glob Public Health. 2026 Mar 26;6(3):e0006087. doi: 10.1371/journal.pgph.0006087 (PMC13020777; doi:10.1371/journal.pgph.0006087)
Supplement: Attachment — Submitted filename: Response to reviewers-Feb13.docx [file pgph.0006087.s007.docx]

February 13, 2026

Dr. Andrew Kazibwe

Academic Editor

PLOS Global Public Health

Dear Dr. Andrew Kazibwe,

Thank you for giving us the opportunity to submit a revised draft of our manuscript titled **The integration of communicable and non-communicable disease (CD-NCD) health services in Africa: A Scoping Review** to *PLOS Global Public Health.* We appreciate the time and effort that you have dedicated to providing valuable feedback on our manuscript.

Attached to this letter, you will find point-by-point responses to comments from the editorial staff. The main changes were:

- We have updated the protocol and noted the update to our Open Science Framework repository
- We completed all necessary formatting revisions to the references.

We believe that the suggested changes have led to an improved manuscript and pushed this work to be both more precise, transparent, and relevant to interested readers. We look forward to discussing this research with the editorial team. Thank you very much for your consideration of our revised manuscript and response in this resubmission.

Best regards,

Takhona Hlatshwako, MSc

PhD Student, Health Policy and Management

University of North Carolina at Chapel Hill

**Comments from Editorial Staff**

1. Please review your reference list to ensure that it is complete and correct. If you have cited papers that have been retracted, please include the rationale for doing so in the manuscript text, or remove these references and replace them with relevant current references. Any changes to the reference list should be mentioned in the rebuttal letter that accompanies your revised manuscript. If you need to cite a retracted article, indicate the article’s retracted status in the References list and also include a citation and full reference for the retraction notice.

Thank you for this reminder. We have reviewed the reference list and corrected formatting errors. No references have been replaced –only edited to align with the journal requirements. We did not find any articles that had been retracted when we reviewed on February 12, 2026. We confirm that the reference list is complete and correct.

Additional Editor Comments (if provided):

2. I thank the authors for addressing my previous comments and for the additional insight on the number of searches conducted. However, this might be perceived as a lack of transparency in the review process. I therefore suggest that the authors revise the PRISMA-ScR diagram to reflect the additional sources that were included in the review after the repeat search conducted in June 2025 and to share a revised review protocol to reflect these changes. This revised protocol should reference the original protocol and replace the original protocol as the guiding document for the review

We appreciate this suggestion. We have revised the PRISMA-ScR diagram to clearly distinguish the preliminary search and repeat search, and when these were conducted. We have also updated the study protocol to reflect these changes, which can be viewed here: <https://osf.io/bctjf/files/54ewn> or on our repository here <https://doi.org/10.17605/OSF.IO/ZMR5K>. The Open Science Framework repository also shows that the protocol has been updated, and viewers can also see the previous version of the protocol to ensure full transparency. We hope that these changes more clearly document our process and the rigor that was employed by conducting two robust searches.
